# Melanogenesis inhibitory activity of Korean *Undaria pinnatifida* in mouse B16 melanoma cells

**DOI:** 10.2478/intox-2014-0012

**Published:** 2014-11-15

**Authors:** Min-Jin Kim, Dong Sam Kim, Hun-Seok Yoon, Wook Jae Lee, Nam Ho Lee, Chang-Gu Hyun

**Affiliations:** 1 Jeju Biodiversity Research Institute (JBRI), Jeju Technopark, Jeju 699-943, Korea; 2 Cosmetic Science Center, Faculty of Chemistry and Cosmetics, Jeju National University, Jeju 690-756, Korea

**Keywords:** *Undaria pinnatifida*, melanin, melanogenesis, tyrosinase, MITF

## Abstract

A number of seaweed species are used as traditional foods and medicine in different parts of the world, including Asian countries. However, very few data on the anti-melanogenic effect of seaweed have been published. *Undaria pinnatifida* (Dolmiyeok), a brown alga, is a traditional food in Jeju Island, the southern regions of the Korea peninsula. In this study, ethylacetate extracts of *U. pinnatifida* (UPE) were examined for their anti-melanogenic potentials. Our results supports the finding that UPE down-regulated melanin content in a dose-dependent pattern. To clarify the target of UPE action in melanogenesis, we performed Western blotting for tyrosinase and microphthalmia-associated transcription factor (MITF), which are key melanogenic enzymes. UPE inhibited tyrosinase and MITF expressions in a dose-dependent manner. These results indicate that treatment with UPE significantly inhibits the melanogenesis in B16 cells, and may be effective in the whitening agent for the skin.

## Introduction

There are many traditional foodstuffs in Korea. *Undaria pinnatifida*, called “Dolmiyeok or Miyeok” is one of them and is considered to be a healthy foodstuff in Korea. It is a very large brown alga, golden-brown in colour, related to the *Laminaria* species and other kelps. Adult specimens can grow to an overall length of between 1.5 and 3 m in less than a year – a rate of growth of up to one centimetre per day. *Undaria pinnatifida* is widely distributed along the coasts of Korea, Japan, China and Russia. Considering the traditional concept, several studies have focused on the beneficial effects of *U. pinnatifida* on anti-inflammatory (Khan *et al.*, 2009; Yoo *et al.*, 2012), anti-oxidant (Han *et al.*, 2004; Hu *et al.*, 2010; Moreira *et al.*, 2010), antihypertensive (Suetsuna *et al.*, 2004), antiviral (Thompson & Dragar, 2004; Hayashi *et al.*, 2013), anticoagulating (Kim *et al.*, 2010), and antiobesity (Okada *et al.*, 2011) activities. However, the anti-melanogenic effect of *U. pinnatifida* extract has not been reported until now. Therefore, we conducted a detailed study to investigate the anti-melanogenic effects of *U. pinnatifida* extract in mouse B16 melanoma cells.

Melanin, produced by melanocytes in the basal layer of the epidermis, is principally responsible for skin colour and plays an important role in preventing skin damage caused by ultraviolet (UV) radiation. Melanin synthesis begins the conversion of L-tyrosine to 3,4-dihydroxyphenylalanine (L-DOPA) and then the oxidation of L-DOPA yields dopaquinone by tyrosinase enzyme, catalyzing the rate-limiting step for the melanin biosynthesis. This tyrosinase enzyme is involved in abnormal accumulation of melanin pigments, called hyperpigmentation (Wu *et al.*, 2012; Chai *et al.*, 2013). Therefore, tyrosinase inhibitors have been established as important constituents of whitening and depigmenting agents (Khan, 2012; Liang *et al.*, 2013). However, side effects have been caused by the chemical inhibitors of tyrosinase. For example, arbutin caused a possible genotoxic effect and kojic acid, due to pigmented contact caused dermatitis. Thus the search for a safe and effective skin whitening agent is still a target of many studies in cosmetic industry (Yoon *et al.*, 2010c; Kim *et al.*, 2013).

A variety of seaweeds provide an interesting, largely unexplored source for the development of potential new drugs and skin-care cosmetics (Yang *et al.*, 2010a;b;d; Yoon *et al.*, 2010a; d). They have existed from antiquity to the present and have played significant roles in skin health and drug discovery, especially anti-inflammatory agents against skin diseases (Moon *et al.*, 2011, Yang *et al.*, 2010c; Yoon *et al.*, 2010b). The seaweed extracts are also known to be inhibitors of melanin production, sometimes more potent than the classical inhibitors hydroquinone/arbutin or kojic acid, and not associated with side effects. Therefore, the present study focused on whether the ethyl acetate fraction from *U. pinnatifida* (UPE) inhibited melanin production and melanogenic protein expression in mouse B16 melanoma cells.

## Materials and methods

### Materials and solvent extraction

*U. pinnatifida* specimens were collected in April 2010 from Gapa Island, Korea. The specimen voucher (no. CSC-201) is deposited with Cosmetic Science Center, Department of Chemistry, Jeju National University, frozen and stored at –20°C until use. For extraction, the material was first ground into a fine powder and freeze-dried using a vacuum freeze-dryer. The dried powder (90g) was extracted with 80% ethanol (EtOH; 2 L) at room temperature for 24 h and then evaporated under vacuum. The evaporated EtOH extract (5g) was suspended in water (1L) and fractionated with ethyl acetate (EtOAc; 500mL). The yield and recovery of EtOAc fractions were 0.6535g and 13.1%, respectively.

### Cell cultures

B16 murine melanoma cells were obtained from the Korean Cell Line Bank (Seoul). Cells were maintained in Dulbecco's modified Eagle's medium (DMEM), supplemented with 10% foetal bovine serum (FBS, Hyclone, Logan, UT, U.S.A.) and 1% penicillin-streptomycin (10,000 U/ml and 10,000 g/ml, respectively) in 5% CO_2_ at 37°C.

### Cell viability assay

Cell viability assay was measured as described previously, with slight modification (Yoon *et al.*, 2010c). Cell viability was determined by the 3-(4,5-dimethylthiazol-2-yl)-2,5-diphenyltetrazolium bromide (MTT) assay. Cells were seeded on 96-well plates, and drug treatment began 18 h after seeding, B16F10 murine melanoma cells were incubated with various concentrations of UPE for 72h at 37°C in a humidified 95% air and 5% CO_2_ atmosphere. MTT (1mg/mL in phosphate-buffer saline, PBS) was added to each well in a 1/10 volume of medium. Cells were incubated at 37°C for 4h. Finally, the supernatant was removed and the formazan crystals were dissolved in DMSO. Absorbance was measured at 570nm. Percent of cells showing cytotoxicity was determined relative to the control group.

### Measurement of melanin contents

Extracellular melanin release was measured as described previously, with slight modification (Yoon *et al.*, 2010c). Cells of the murine melanoma cell line, B16F10, plated at 1.0×10^5^ cells/mL, were stimulated with α-MSH (50nM) and then incubated with aliquots of UPE (3.125, 6.25 and 12.5µg/mL) at 37°C for 72h; the cells were then washed in ice-cold phosphate-buffered saline. Briefly, the samples were incubated at 80°C for 1h in 1mL of 1N NaOH/10% DMSO and then vortexed to solubilize the melanin; the absorbance at 405nm was then measured. Further, the melanin content was determined based on the absorbance/µg of protein in the extract from each cell. The protein concentration of the cells was determined by using a protein assay kit (Pierce, Rockford, IL, USA).

### Determination of cellular tyrosinase activity

Cellular tyrosinase activity was measured by the method of Yen *et al.* with some modification (Yen *et al.*, 2012). Briefly, the culture method for determining cellular tyrosinase assay was similar to that for determining melanin content. After treatment with different concentrations of UPE for 72 h, the cells were collected after treatment with trypsin-EDTA and centrifuged at 15,000 rpm for 15 min to obtain cell pellets. The pellet solutions were frozen and thawed twice and then centrifuged at 15,000 rpm for 15 min. We added 80µL of the supernatant in a 96-well plate and mixed it with 20µL of 0.2% L-DOPA. After incubation for 1h, the optical densities were measured at 475nm using a microplate spectrophotometer. The inhibitory activity of the UPE treated cells was presented as percentage against that of the untreated cells.

### Measurement of tyrosinase and MITF in melanoma B16/F10 cells by Western blot

To determine the amount of tyrosinase and MITF protein, Western blotting analysis was performed. B16 melanoma cells that had been stimulated by α-MSH (50nM) were treated with UPE (3.125, 6.25 and 12.5 µg/mL) for 3 days. After treatment, the cells were collected and lysed with cell lysis buffer [50mM Tris–HCl (pH 6.8), 2% SDS, 6% mercaptoethanol, 1% glycerol]. Whole cell lysates (5×10^4^ cells equivalents per lane) were separated by 7.5% SDS-polyacrylamide gel electrophoresis as described previously and transferred to a polyvinylidene fluoride (PVDF) membrane. The membrane was blocked with 5% skimmed milk in phosphate-buffered saline containing 0.05% Tween 20. Tyrosinase and MITF bands were detected with rabbit polyclonal anti-tyrosinase antibody (dilution 1:1000) and rabbit polyclonal anti-MITF antibody (dilution 1:500), respectively, purchased from Santa Cruz Biotechnology (Santa Cruz, CA, USA), and then further incubated with horseradish peroxidase-conjugated anti-rabbit IgG antibody at a 1:5000 dilution. Bound antibodies were detected using an enhanced chemiluminescence kit (Amersham Biosciences, Buckinghamshire, UK), following the manufacturer's instructions. Loading control was assessed using anti-β-actin antibody. Positive bands were analysed using a gel image analysis instrument.

### Statistical analysis

All data were obtained in triplicate and are represented as means ± standard error (SE). Significant differences between treatments were determined by Student's *t* test in one-way analysis of variance (ANOVA).

## Results and discussion

**Figure 1:**
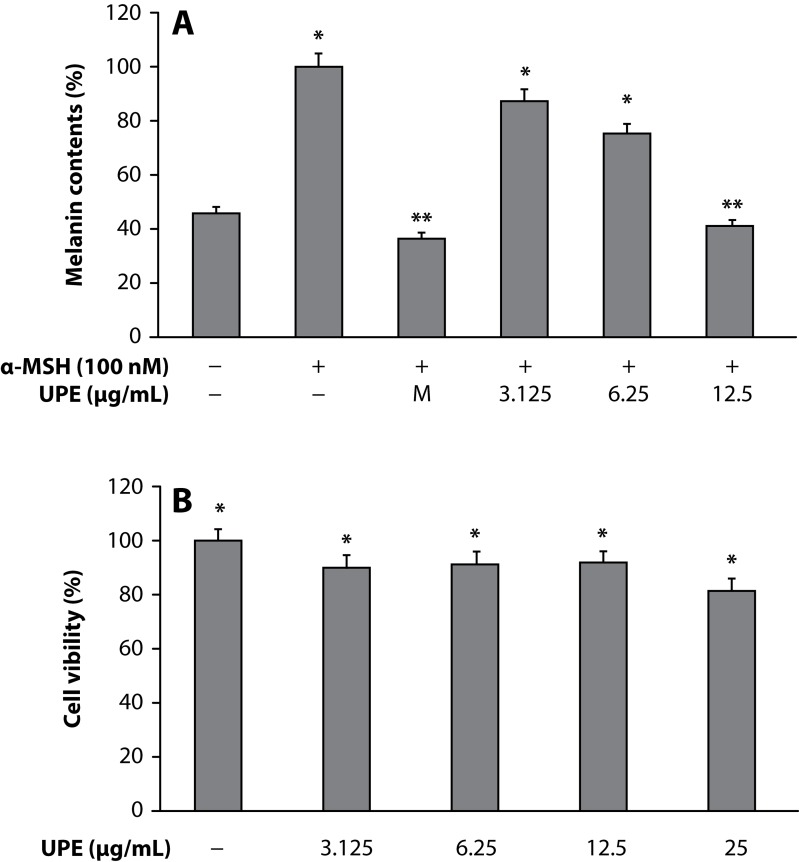
Inhibitory effect of UPE on melanin content (A) and cell viability (B) of B16F10 cells. B16F10 cells (2.0×10^4^ µg/mL) were pre-incubated for 18 h and the melanin content was assayed after incubation of the B16F10 cells treated with α-MSH (100 nM), melasolv (40 µM), and UPE (3.125, 6.25 and 12.5 µg/mL) for 72 h at 37°C in a 5% CO_2_ atmosphere. The absorbance was measured at 405 nm by ELISA. MTT assay was performed after incubation of the B16F10 cells treated with varying concentrations of UPE (3.125, 6.25 and 12.5 µg/mL) for 24 h at 37°C in a 5% CO_2_ atmosphere. The absorbance was measured at 570 nm with a spectrophotometer (Power Wave; Bio-tek, Winooski, VT). Values are the mean ± SEM of triplicate experiments. *p<0.05; **p<0.01.

**Figure 2:**
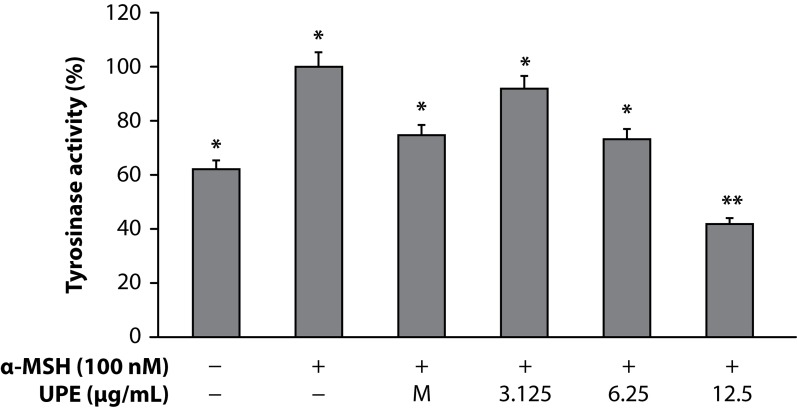
Inhibitory effect on tyrosinase activity of UPE in B16F10 cells. B16F10 cells (2.0×10^4^ µg/mL) were pre-incubated for 18 h and tyrosinase activity was assessed after incubation of B16F10 cells treated with α-MSH (100 nM), melasolv (40 uM) and UPE (3.125, 6.25 and 12.5 µg/mL) for 72 h at 37°C in a 5% CO_2_ atmosphere. Absorbance was measured at 405 nm with a ELISA. Values are the mean ± SEM of triplicate experiments.*p<0.05; **p<0.01.

Melanin plays a crucial role in protecting the skin against harmful ultraviolet light, but overproduction and accumulation of melanin could create serious skin problems such as freckles, age pigment, and melasma. Thus, the inhibition of melanogenesis has been the focus on medicinal and cosmetic treatments for skin depigmenting and lightening. Therefore, this study focused on whether the ethyl acetate fraction from UPE inhibited melanin production and melanogenic protein expression in mouse B16 melanoma cells. In the present study, the changes in the melanin contents in the cells treated with UPE were evaluated for anti-melanogenesis activity. The melanin contents of cells were significantly attenuated by UPE in a dose-dependent manner (Figure 1A). In spite of a number of studies reporting on anti-melanogenic agents, such as hydroquinone, kojic acid, and arbutin, sometimes side effects such as irritation of the skin and exhibition of cell toxicity were observed. Therefore it is necessary to find potent natural products that act as anti-melanogenic agents without side effects. To investigate the cytotoxicity of UPE on cell proliferation, B16 murine melanoma cells were treated with various concentrations (3.125–25µg/mL) of UPE for 72h. As shown in Figure 1B, there was no significant difference in cell proliferation between control and UPE-treated cells until 12.5 µg/mL, suggesting that the inhibitory effects of UPE on melanin biosynthesis were not attributable to its cytotoxicity. Since cellular tyrosinase activity is also the major factor that stimulates melanin synthesis and ultimately induces melanogenesis, we determined to assess cellular tyrosinase activity for investigating the antimelanogenesis activity of UPE on B16 murine melanoma cells. B16 murine melanoma cells were pretreated with UPE at doses of 3.125–12.5µg/mL. UPE treatment significantly reduced the cellular tyrosinase activity in a dose-dependent manner compared to the control (Figure 2).

**Figure 3:**
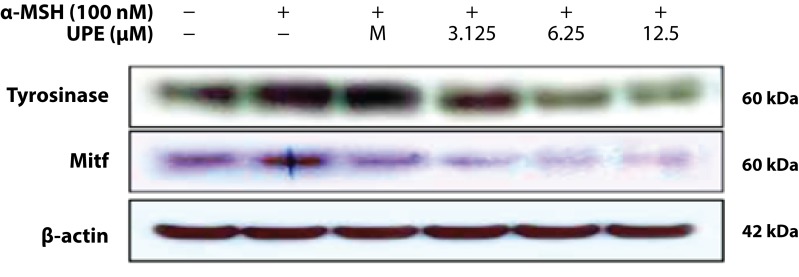
Inhibitory effect of the UPE on the protein level related to melanogenic factors in B16F10 cells. B16F10 cells (1.0×10^5^cells/mL) were pre-incubated for 18 h and were stimulated with α-MSH (100nM) in the presence of melasolv (40 µM) and UPE (3.125, 6.25 and 12.5 µg/mL) for 24 h. The protein level was determined by immunoblotting.

Human melanocytes are known to express tyrosinase and MITF is a factor that effectively transactivates tyrosinase. The expression of these proteins was evaluated using Western blot analysis. As compared with the untreated control values, UPE-treated cells showed dose-dependent decreases in tyrosinase and MITF expression (Figure 3).

In summary, we investigated the anti-melanogenic effects of UPE and related melanogenic activity. The present results suggest that MITF protein levels are reduced by UPE. The hypopigmentation effect of UPE may be the result of down-regulation of MITF gene expression, which would then repress the protein and gene expressions of tyrosinase. Therefore we suggest that UPE can be a useful inhibitor of melanogenesis and has beneficial effects in the treatment of hyperpigmentation disorders such as ephelis and oedema. However, the inhibitory mechanism of melanin production in B16 murine melanoma cells by UPE remains unclear. The investigation of the exact mechanisms and further *in vivo* experiments are needed to evaluate the possible use of UPE as a natural skin-whitening agent.
